# Vascular Endothelial Growth Factor and Brain-Derived Neurotrophic Factor in Quetiapine Treated First-Episode Psychosis

**DOI:** 10.1155/2014/719395

**Published:** 2014-02-05

**Authors:** Brendan P. Murphy, Terence Y. Pang, Anthony J. Hannan, Tina-Marie Proffitt, Mirabel McConchie, Melissa Kerr, Connie Markulev, Colin O'Donnell, Patrick D. McGorry, Gregor E. Berger

**Affiliations:** ^1^Early in Life Mental Health Service, Southern Health, Melbourne, VIC 3175, Australia; ^2^School of Psychology and Psychiatry, Monash University, Melbourne, VIC 3800, Australia; ^3^Howard Florey Institute, Florey Neuroscience Institutes, University of Melbourne, Melbourne, VIC 3052, Australia; ^4^Orygen Youth Health Research Centre, University of Melbourne, Melbourne, VIC 3052, Australia; ^5^St. Davnet's Hospital, Monaghan, County Monaghan, Ireland; ^6^Department of Child and Adolescent Psychiatry, University of Zurich, 8032 Zurich, Switzerland

## Abstract

*Objective*. It has been suggested that atypical antipsychotics confer their effects via brain-derived neurotrophic factor (BDNF). We investigated the effect of quetiapine on serum levels of BDNF and vascular endothelial growth factor (VEGF) in drug-naive first-episode psychosis subjects. *Methods*. Fifteen patients drawn from a larger study received quetiapine treatment for twelve weeks. Baseline levels of serum BDNF and VEGF were compared to age- and sex-matched healthy controls and to levels following treatment. Linear regression analyses were performed to determine the relationship of BDNF and VEGF levels with outcome measures at baseline and week 12. *Results*. The mean serum BDNF level was significantly higher at week 12 compared to baseline and correlated with reductions in Brief Psychiatric Rating Scale (BPRS) and general psychopathology scores. Changes in serum VEGF levels also correlated significantly with a reduction in BPRS scores, a significant improvement in PANNS positive symptoms scores, and displayed a positive relationship with changes in BDNF levels. *Conclusions*. Our findings suggest that BDNF and VEGF are potential biomarkers for gauging improvement of psychotic symptoms. This suggests a novel neurotrophic-based mechanism of the drug effects of quetiapine on psychosis. This is the first report of VEGF perturbation in psychosis.

## 1. Introduction

The precise molecular mechanisms mediating the actions of antipsychotic drugs remain poorly understood [1]. It has been proposed that atypical antipsychotics confer some of their benefits through neuroprotective effects [2]. Evidence from rodent studies suggests that this protective effect is mediated by an upregulation of brain-derived neurotrophic factor (BDNF) in the brain [3–5]. A review of animal studies concluded that, with the exception of risperidone, atypical antipsychotics might increase BDNF expression while conventional antipsychotics might decrease expression [6]. Risperidone's inability to alter expression of BDNF or to even increase it at higher doses (Favelli) suggests differential effects of atypical antipsychotics on BDNF expression. Olanzapine and risperidone significantly increase D2 binding in the medial prefrontal cortex and hippocampus, whilst quetiapine does not. Similarly, olanzapine and risperidone produce significant up-regulation of D4 receptors in the hippocampus, caudate-putamen, and nucleus accumbens, whilst quetiapine does not [7]. Quetiapine's inability to adjust dopamine receptors suggests that nondopaminergic mechanisms may contribute more to its efficacy than with other atypical antipsychotics. Quetiapine has been shown to attenuate stress-induced reductions in BDNF expression in rats [5].

A previous study of drug-naive first-episode psychosis patients reported that serum BDNF levels are lower in this patient group and were increased toward healthy control levels following olanzapine treatment, in association with a reduction of positive symptoms [8].

*Brain-Derived Neurotrophic Factor*. BDNF is the most widely distributed neurotrophin in the central nervous system (CNS) [9] and is highly expressed in the prefrontal cortex and hippocampus [10]. It serves a variety of essential functions during development including neurogenesis, neuronal differentiation and survival, and normal maturation of neurodevelopmental pathways [11, 12]. In the adult, it continues to play a crucial role in neuronal integrity, promoting synaptic plasticity [13] as well as modulating synthesis, metabolism, and release of dopamine, GABA, serotonin and glutamate [14] and moderating use-dependent plasticity processes including long-term potentiation, learning, and memory [15, 16]. Perturbations in BDNF are likely to have widespread psychopathological consequences leading to altered brain development and functioning [17], and there is accumulating evidence that abnormal BDNF signalling may be involved in the pathophysiology of a range of psychiatric disorders including depression [18], bipolar affective disorder [19], and schizophrenia [20].

There are several lines of evidence pointing to a possible role in schizophrenia. Firstly, BDNF is known to be important both in the neurodevelopment of dopaminergic-related systems and in ongoing dopaminergic function [21], particularly exerting its effects through the mesolimbic pathway [22]. Secondly, as mentioned, BDNF regulates other neurotransmitters putatively involved in the aetiology of schizophrenia, including glutamate, GABA, and serotonin [14]. Thirdly, BDNF principally exercises its influence through neuroanatomical areas known to be associated with symptom domains in schizophrenia, including the prefrontal cortex and hippocampus [10].

Evidence for disturbed BDNF functioning in schizophrenia comes from several different sources. Several studies have found alterations in postmortem levels of BDNF in the brains of schizophrenic subjects: decreased levels in the prefrontal cortex [23–25] and hippocampus [26] and increased levels in the hippocampus [27, 28], anterior cingulate cortex [28], and cerebral cortex [26]. Another study reported deceased gene expression of BDNF and TrkB receptors in postmortem schizophrenic brains [29]. BDNF is known to mediate its effects through binding to TrkB receptors, which in turn activate intracellular survival pathways [17].

BDNF crosses the blood-brain barrier [30] and serum concentrations strongly correlate with brain levels [31]. Reports of BDNF levels in the serum of schizophrenic patients have proven inconsistent. The majority demonstrate a significant reduction in serum BDNF in chronic and medicated schizophrenics compared to healthy controls [32–38], though there have been reverse findings [39–41]. Similarly, amongst first-episode and drug-naive cohorts, significant reductions relative to healthy subjects have been reported more often [42–49] than not [50–53]. Unlike postmortem samples or chronic cohorts, FEP populations—especially drug-naïve ones—are beneficial to study as the effects of chronicity, medication, and medical comorbidities are attenuated. A recent systematic review and meta-analysis concluded that there was moderate evidence for reduced serum BDNF levels in medicated and drug-naive schizophrenia, with an association between reduced levels and increasing age, but no effects of medication dosage [54]. However, the reviewers were unable to account for the heterogeneity across studies, though gender and body mass are known to affect BDNF levels, as are stage of illness, subtype of schizophrenia and medication [52], substance misuse [53] and diet [54].

*Vascular Endothelial Growth Factor*. The cytokine vascular endothelial growth factor (VEGF) is critical for blood vessel growth during embryonic development and in adults, promoting vasculogenesis and playing a central role in wound healing [55]. As well as indirectly promoting neurogenesis by regulating brain angiogenesis, VEGF is also believed to have direct trophic effects on neurons and glia in the CNS, supporting neuronal migration in the developing CNS and displaying essential neuroprotective properties in the adult. While VEGF is believed to have significant effects on hippocampal morphology [56] and its role in treating depression has been explored [57], there has been little research with respect to schizophrenia. There has been one study that reported decreased VEGF mRNA levels in the dorsolateral prefrontal cortex of schizophrenic subjects [58]. In rats, VEGF levels are increased in the hippocampus after 14 days of treatment with haloperidol and olanzapine. Upon continuing with daily drug administration, it was found that the effect of haloperidol was lost after 45 days of treatment while VEGF levels continued to rise in the olanzapine-treated rats. That difference was suggested to reflect the increased efficacy in treating cognitive and negative symptoms that second generation antipsychotics demonstrate [59]. In support of this, chlorpromazine has been shown to reduce VEGF secretion *in vitro* [60].

VEGF levels have not been measured in schizophrenic subjects though there have been several studies in depression. One study found raised plasma VEGF levels in 16 depressed subjects [61] while another found raised levels in 35 depressed subjects and 35 manic subjects compared to healthy controls. The latter study also reported that there was no correlation of VEGF levels to symptoms and the authors suggested that the raised levels may be serving a neuroprotective function [62]. VEGF levels were not significantly altered by antidepressant therapy in 25 depressed subjects [63], though one study demonstrated significant increases in 19 depressed subjects one month after ECT treatment that correlated with an improvement in depression scores [64].

*Aims*. There have been no previous studies investigating the effects of quetiapine on serum BDNF levels in FEP. We therefore sought to compare baseline BDNF levels in drug-naive FEP subjects with age- and sex-matched healthy controls and then to determine whether there was a change in levels in FEP subjects following 12 weeks of treatment with quetiapine. We hypothesised that baseline subjects would have lower BDNF levels than healthy controls but that levels would improve with treatment. We also hypothesised that there would be a negative correlation between BDNF levels and a number of outcome measures, including positive and negative symptoms.

There has been no exploration of VEGF in schizophrenia or other psychoses. We therefore sought to measure VEGF levels in our sample. We hypothesised that baseline subjects would have lower VEGF levels than healthy controls, which would increase following quetiapine treatment. We also hypothesized that changes to VEGF levels would be negatively correlated to a number of outcome measures. Finally, we proposed that there would be a positive correlation between BDNF and VEGF levels.

## 2. Materials and Methods

### 2.1. Subjects

15 subjects (10 males/5 females; mean age = 18.6 ± 3.3 years) were drawn from a prospective, randomized, double-blind cohort study (Trial Registration: clinicaltrials.gov Identifier: NCT00449397) that compared 4-weeks of 200 mg/day versus 400 mg/day of quetiapine for efficacy, tolerability, and safety in 141 drug-naive first-episode psychosis patients aged 15–25 [65]. All subjects who had provided sufficient samples at baseline and week 12 had their serum assayed. Eight subjects were completely drug-naive, two subjects had received 3 days of quetiapine, four had received 4 days of quetiapine, and one had received 7 days of quetiapine prior to serum collection. Five subjects were randomized to 200 mg, ten to 400 mg. The control group consisted of a group of fifteen healthy volunteers with no history of psychiatric disorder (18.5 ± 3.1 years). Subjects were age- and sex-matched to controls because of the known effects of age and gender [66]. The serum for all subjects was separated in a centrifuge and stored at −80°C until assayed. A first episode of psychosis was defined as daily psychotic symptoms for longer than a week that could not be explained by other factors (e.g., organic in the context of temporal lobe epilepsy or drug-induced psychotic episodes that remitted within 7 days without antipsychotic medication. Subjects were recruited from the Early Psychosis Prevention and Intervention Centre (EPPIC), a subprogram of Orygen Youth Health [67]. Exclusion criteria included having received more than one week of previous treatment with an antipsychotic, a concurrent manic episode, and a history of neurological disease. Subjects were randomly allocated to either 200 mg or 400 mg of quetiapine and treated for four weeks. Following this, subjects remained on quetiapine for a further eight weeks and had their doses adjusted according to clinical need. No other antipsychotic was permitted during the twelve week study. Subjects were assessed using SCID-IV, Research Version, Patient Edition (2002), and symptomatology ratings included the BPRS extended version 4, the Scale for the Assessment of Negative Symptoms (SANS) and the Positive and Negative Syndrome Scale (PANSS). Adverse events were assessed using the Udvalg for Kliniske Undersogelser (UKU). All raters undertook systematic psychopathology training in the context of annual rater workshops at the Orygen Research Centre; the ratings demonstrated good reliability, with intraclass coefficients of agreement ≥0.8, and all were within 20% of the standard scores. All raters were blind to BDNF levels. The research was approved by the relevant Research and Ethics Committees and both subjects and controls provided written informed consent.

### 2.2. BDNF Measurement

BDNF concentrations of serum samples were quantified using the BDNF Emax Immunoassay system (Promega, Madison, WI, USA) according to manufacturer's instructions. Briefly, each sample was diluted with sample buffer to obtain two dilutions (1/10 and 1/50) and each was assayed in triplicate. Technical replicates with greater than 10% variance were repeated. After adjusting for dilution factors, samples with greater than 25% variance were repeated. The average serum concentration of BDNF of both dilutions was determined and used for subsequent data analysis.

### 2.3. VEGF Measurement

VEGF concentrations of serum samples were quantified using MILLIPLEX MAP human cytokine/chemokine panel (Cat. number MPXHCYTO-60 K, Millipore, Billerica, MA, USA) in conjunction with a Luminex 100 system (Austin, TX, USA). Undiluted samples were assayed in duplicate and technical replicates with greater than 10% variance were repeated. The serum concentration of VEGF from both readouts was averaged and used for subsequent data analysis.

### 2.4. Statistical Analyses

Data was analysed using GraphPad Prism Version 6.1 (GraphPad Software, La Jolla, California, USA). Data sets were separated according to sex and drug dosage for analysis by one-way ANOVA using Bonferroni adjusted *α*-values of 0.0167. 95% confidence intervals were calculated and *r* ^2^ values determined.

## 3. Results

### 3.1. BDNF

Serum BDNF levels of males and females in the control group were similar, and this was also observed in patient groups at week 0 and week 12 (data not shown). Therefore, sexes were pooled for subsequent analyses. There was no other significant effect of quetiapine dose on BDNF levels in the patient group (data not shown) so both dosages were pooled for subsequent analyses.

**Figure 1:**
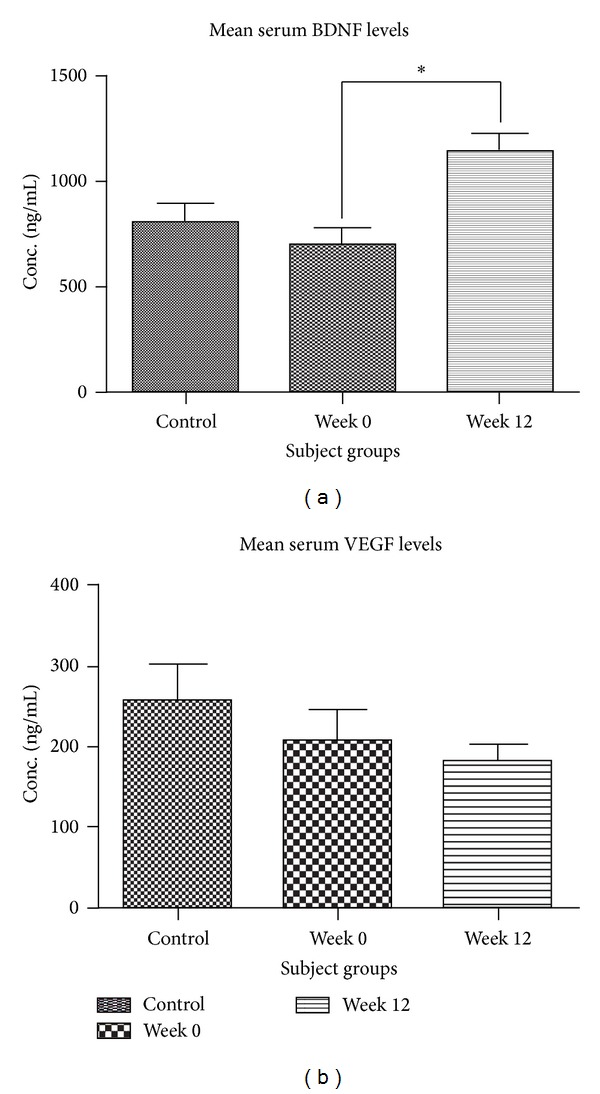
Serum neurotrophin levels. Grouped analysis of serum BDNF levels (a) revealed a greater mean BDNF level of the FEP patient group at week 12 compared to week 0. There was no overall difference in VEGF levels between the three subject groups (b). *One-way ANOVA followed by post hoc *t*-test *P* < 0.05.

One-way ANOVA found a significant difference in the mean serum BDNF levels between the groups (*F*_(2,51)_ = 7.797; *P* = 0.0011; Figure 1(a)), and post hoc testing revealed that this was significantly higher in the Week 12 patient group compared to baseline (Wk12 versus Wk0: *P* = 0.012) but not compared to the control group (*P* = 0.0256).

Linear regression analysis found no significant relationship between Week 0 serum BDNF levels and BPRS, UKU, or SANS scores (see *Supplementary Figures 1(a)*–*1(c)* available online at http://dx.doi.org/10.1155/2014/719395). There was also no relationship between serum BDNF levels and PANSS negative, positive, or general psychopathology scores at Week 0 (see *Supplementary Figures 1(d)*-*1(e)*). At Week 12, there was no significant relationship between serum BDNF levels and outcome scores (see *Supplementary Figure 2*).

**Figure 2:**
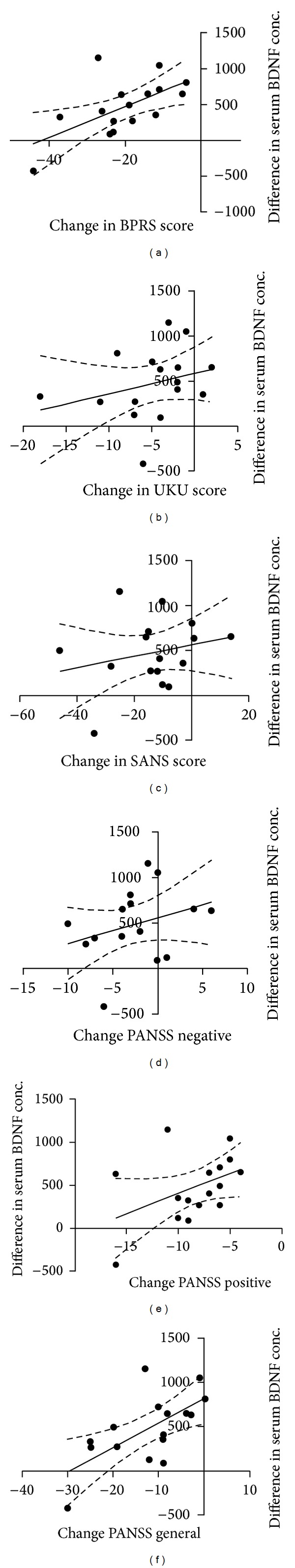
Relationship between changes in serum BDNF concentrations with changes in outcome measures. There were significant positive correlations between serum BDNF levels and improved scores on the BPRS (a) and PANNS general (f). There were no significant correlations for changes in scores of UKU (b), SANS (c), PANSS negative (d), and PANSS positive (e) with the difference in BDNF levels across both time points.

The difference in serum BDNF levels at weeks 0 and 12 was determined and analysed against the change in evaluative test scores. There was a significant positive relationship with reduction in BPRS scores and change in BDNF levels (*r* ^2^ = 0.3656, *P* = 0.0131) (Figure 2(a)), though there was no significant relationship between the change in serum BDNF levels and changes in UKU (*r* ^2^ = 0.08078, *P* = 0.2861; Figure 2(b)) or SANS scores (*r* ^2^ = 0.0569, *P* = 0.3637; Figure 2(c)).

The extent of the reduction in PANSS general psychopathology scores displayed a positive relationship with the change in BDNF levels (*r* ^2^ = 0.4176, *P* = 0.0068; Figure 2(d)) but there was no significant relationship between the change in BDNF levels and change in PANSS positive (*r* ^2^ = 0.1867, *P* = 0.0946; Figure 2(e)) or negative scores (*r* ^2^ = 0.1037, *P* = 0.2239; Figure 2(f)).

### 3.2. VEGF

Mean serum VEGF levels of males and females in all three groups (control group, T0, and T12) did not differ significantly (data not shown); therefore sexes were pooled for subsequent analyses. There was no significant difference in VEGF levels between patients allocated to dosage 1 and dosage 2 at both week 0 and week 12 timepoints and patient data for both dosages were pooled for subsequent analyses. One-way ANOVA analysis of the pooled data revealed no significant difference in the mean serum VEGF levels (*F*_(2,45)_ = 1.095, *P* = 0.3437; Figure 1(b)).

**Figure 3:**
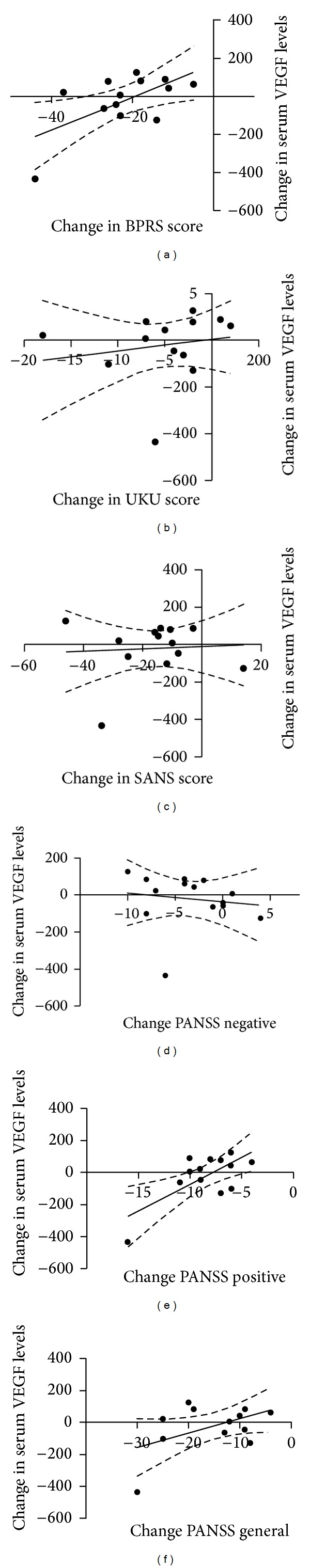
Relationship between changes in serum VEGF concentrations with changes in outcome measures. There were significant positive correlations between serum VEGF levels and improved scores on the BPRS (a) and PANNS positive (d). There were no significant correlations for changes in scores of UKU (b), SANS (c), PANSS negative (d), and PANSS general (f) with the difference in VEGF levels across both time points.

**Figure 4:**
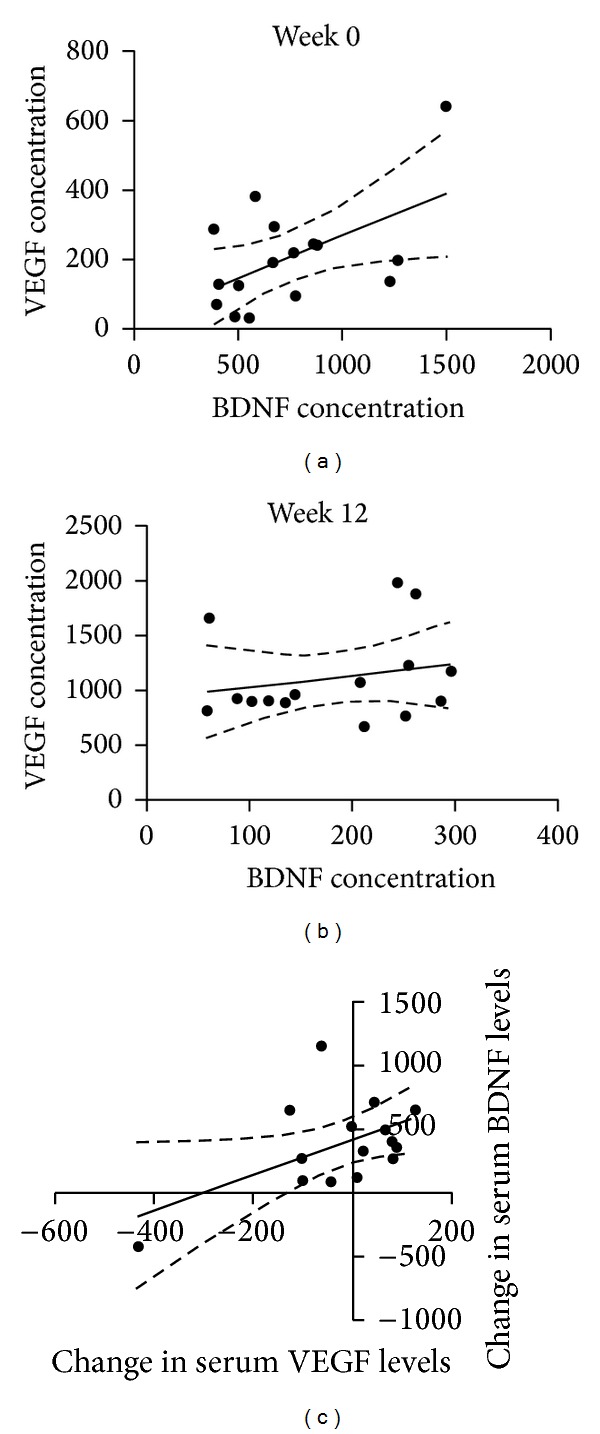
Relationship between BDNF and VEGF levels. Correlation analysis of serum BDNF and VEGF levels at separate time points reveals a significant relationship at week 0 (a) but not at week 12 (b). Further analysis found a significant correlation between the differences in BDNF and VEGF levels (c).

Linear regression analysis found no significant relationship between Week 0 serum VEGF levels and BPRS, UKU, or SANS scores (*Supplementry Figures 3(a)*–*3(c)*). There was also no relationship between serum BDNF levels and PANSS negative, positive, or general psychopathology scores at Week 0 (*Supplementry Figures 3(d)*–*3(f)*). Similarly, at Week 12, there was no significant relationship between serum VEGF levels and outcome scores (*Supplementry Figure 4*).

The difference in serum VEGF levels at weeks 0 and 12 was determined and analysed against the change in evaluative test scores. There was a significant positive relationship with reduction in BPRS scores and change in VEGF levels (*r* ^2^ = 0.3820, *P* = 0.0244; Figure 3(a)), though there was no significant relationship between change in UKU (*r* ^2^ = 0.03133, *P* = 0.5629; Figure 3(b)) or SANS scores (*r* ^2^ = 0.0035, *P* = 0.8473; Figure 3(c)) and the change in serum VEGF levels. The change in VEGF levels displayed a significant positive relationship with the reduction in PANSS positive symptom scores (*r* ^2^ = 0.4847, *P* = 0.0082; Figure 3(d)). However there was no significant relationship between the change in VEGF levels and change in PANSS negative (*r* ^2^ = 0.01708, *P* = 0.6704; Figure 3(e)) or general scores (*r* ^2^ = 0.2457, *P* = 0.0849; Figure 3(f)).

### 3.3. Relationship between BDNF and VEGF

We examined the data set for a possible association between BDNF and VEGF levels. Linear regression revealed that at Week 0, VEGF levels were significantly associated with BDNF levels (*r* ^2^ = 0.2833, *P* = 0.0338; Figure 4(a)) but this relationship was lost at the Week 12 timepoint (*r* ^2^ = 0.04886, *P* = 0.4285; Figure 4(b)). Furthermore, the change in BDNF levels from Week 0 to Week 12 was significantly associated with changes in VEGF levels (*r* ^2^ = 0.2758, *P* = 0.0444; Figure 4(c)).

## 4. Discussion

This study has uncovered a correlative relationship between serum BDNF and VEGF levels in a drug-naive FEP sample. Each was differentially altered by twelve weeks of quetiapine treatment and was associated with specific measures of psychopathology. We believe this is the first study reporting on serum VEGF levels in first episode psychosis. The inverse relationship of serum BDNF levels with BPRS and PANSS general scores raises the possibility of its use in gauging effective antipsychotic treatment for patients in the absence of psychiatric evaluation. The study was limited by the relatively small number of enrolled subjects who had provided both baseline and Week 12 blood samples; however, a high attrition rate and loss to followup are widely acknowledged as two significant challenges facing studies of this nature. Week 12 blood samples from the healthy control group were unavailable so we cannot definitively conclude that our observations are solely attributable to quetiapine treatment. There was also an imbalance in the male/female numbers so we were unable to further examine the trend for males receiving the higher dose of quetiapine to have higher BDNF levels. Finally, full interpretation of our findings is hindered by the lack of knowledge regarding the function of BDNF and VEGF in peripheral circulation.

We found significantly raised BDNF levels following three months of treatment with quetiapine. Furthermore, no significant difference was found between Week 12 levels and controls suggesting that BDNF levels normalise with treatment. While there was no correlation between the absolute serum concentration and outcome measures at baseline or week 12, we found that the change in serum BDNF levels was positively correlated with specific reductions in BPRS and PANSS general psychopathology scores. No association was found with PANNS positive, negative, SANS, or UKU scores. It is known that negative symptoms take longer to respond to treatment so it is possible that with extended treatment beyond 12 weeks, changes in the other outcome measures could display a relationship with BDNF levels. This could be the focus of follow-up studies.

The few other studies that have attempted to correlate serum BDNF levels with outcome measures in FEP have had mixed results. In a study of 15 FEP subjects, a negative correlation between baseline BDNF levels and positive symptom scores was found but not with negative symptoms scores or any cognitive or motor function scores [42]. In two separate studies, Rizos et al., found negative correlations between baseline BDNF levels and both positive and negative symptom scores in 14 FEP subjects [48] and to the duration of untreated psychosis in 37 drug-naive FEP subjects [47]. Only one other published FEP study has correlated serum BDNF levels with outcome scores *after* treatment with antipsychotics. González-Pinto et al. (2010) found that serum BDNF levels were correlated to functional outcome and improved positive symptoms, though not to negative symptoms or general psychopathology in 11 olanzapine treated FEP subjects [44]. That study reported raised serum BDNF levels following treatment with antipsychotics, as have three other FEP studies (unspecified treatment [46]; aripiprazole [46]; risperidone, olanzapine, and aripiprazole [53]) which are consistent with the findings of this study. Amongst chronic cohorts, risperidone [47, 53], amisulpride [47], and haloperidol [47] did not raise BDNF levels, whilst clozapine [32, 68] and olanzapine [47] did. Collectively, there is some evidence that outcome measures are associated with serum BDNF levels, regardless of whether that relationship is direct or indirect; however, further studies involving larger cohorts of subjects and incorporating multiple antipsychotic drugs will be required before a more definitive conclusion can be reached.

Besides outcome measures, serum BDNF levels have been positively correlated with right hippocampal volumes in 20 drug-naive FEP subjects, with subjects having smaller hippocampi than controls [69]. There has been extensive research over the past decade examining the influence of the val66met single nucleotide polymorphism (SNP) of the BDNF gene [70] and its association with psychiatric conditions including schizophrenia, anorexia nervosa [71], and obsessive-compulsive disorder [72]. While young healthy carriers of the val66met polymorphism have smaller hippocampi, older healthy carriers do not, although this disparity could be due to an age-by-allele interaction since both BDNF expression and hippocampal volumes decrease with age [73]. Studies in schizophrenic subjects have reported mixed findings, one study finding smaller volumes with the met polymorphism [74], another smaller volumes with val homozygotes [75], and three finding no association [76–78]. A recent study in first-episode psychosis (FEP) patients found no difference in hippocampal volumes between healthy met controls and FEP met carriers but reduced volumes for FEP val homozygotes compared to healthy val controls. It would be interesting to probe our dataset for associations between BDNF, VEGF, and hippocampal volumes.

Beyond the hippocampus, others have found a relationship between the met polymorphism, temporal, and occipital lobe volume reductions and cognitive impairment [79], as well as reduced frontal lobe volumes in FEP met carriers compared to val homozygotes [80]. While the SNP has not been shown to influence response to treatment [81], it has been associated with an earlier age of onset of symptoms [82] and the development of tardive dyskinesia [3, 83]; though there have been negative findings [84, 85], the positive reports complementing the findings of lower serum BDNF levels amongst those with tardive dyskniesia [86, 87].

Though there was a trend for males on 400 mg of quetiapine to have higher BDNF levels at T12, no correlation was found between dose of quetiapine and BDNF levels. While this may have been due to low sample size or the fact that the doses were too similar (and doses in both groups were increased from week 4 to 12 according to clinical need), the only other FEP study to correlate antipsychotic dose with serum BDNF found no correlation with aripiprazole dose [49]. This accords with the aforementioned meta-analysis [54], though clozapine dose does correlate with serum BDNF in chronic schizophrenia [32, 68].

To our knowledge this is the first report that serum BDNF levels are increased by treatment with quetiapine in subjects with psychosis and complements the other positive findings of raised BDNF with atypical antipsychotic treatment and improvement in outcome scores following treatment. It also suggests a direct neurotrophic role for quetiapine via raising BDNF. Quetiapine has been shown to decrease the stress-induced reduction in BDNF expression in rat hippocampus [5], increase BDNF mRNA expression in rat hippocampus [88], and reverse the stress-induced suppression of rat hippocampal neurogenesis [89]. Taken together with our findings, these results suggest that quetiapine has a direct neurotrophic effect in schizophrenia, promoting neuroplasticity via the up-regulation of BDNF.

We also found that VEGF levels at baseline were significantly associated with BDNF levels, though this relationship was lost by Week 12. Nevertheless, the change in serum BDNF levels from Week 0 to Week 12 was associated with a change in serum VEGF levels. Furthermore, as with BDNF, the increase in serum VEGF positively correlated with a reduction in both BPRS and PANSS positive symptom scores. This is the first study to measure serum VEGF levels in psychosis and the findings are extremely novel. The correlation between increases in VEGF and BDNF and their similar impact on BPRS and general psychopathology scores suggests a direct neurotrophic role for VEGF, independent of secondary neurogenesis via brain angiogenesis. The findings also suggest that any direct neurotrophic effect quetiapine possesses maybe via several pathways including promoting BDNF and VEGF expression. Finally, it raises the possibility of gauging improvement of psychotic symptoms based by measuring changes in serum levels of BDNF and VEGF.

## 5. Conclusion

Our study investigated the peripheral levels of two key neurotrophic factors and report on their differential regulation by quetiapine treatment. Our results revealed an intriguing prospect that increases of serum BDNF levels might reflect the positive behavioural effects of antipsychotic treatment. Further research will be required to validate this as well as to examine if other antipsychotic agents similarly moderate VEGF levels.

## Supplementary Material

Linear regression analyses of serum BDNF and VEGF levels with the various psychiatric evaluative assessments.
