# Supplementary material for: Vascular Endothelial Growth Factor and Brain-Derived Neurotrophic Factor in Quetiapine Treated First-Episode Psychosis
Source: Schizophr Res Treatment. 2014 Feb 5;2014:719395. doi: 10.1155/2014/719395 (PMC3941155; doi:10.1155/2014/719395)
Supplement: Supplementary file 1 — Linear regression analyses of serum BDNF and VEGF levels with the various psychiatric evaluative assessments. [file 719395.f1.pdf]

Supplementary Figures

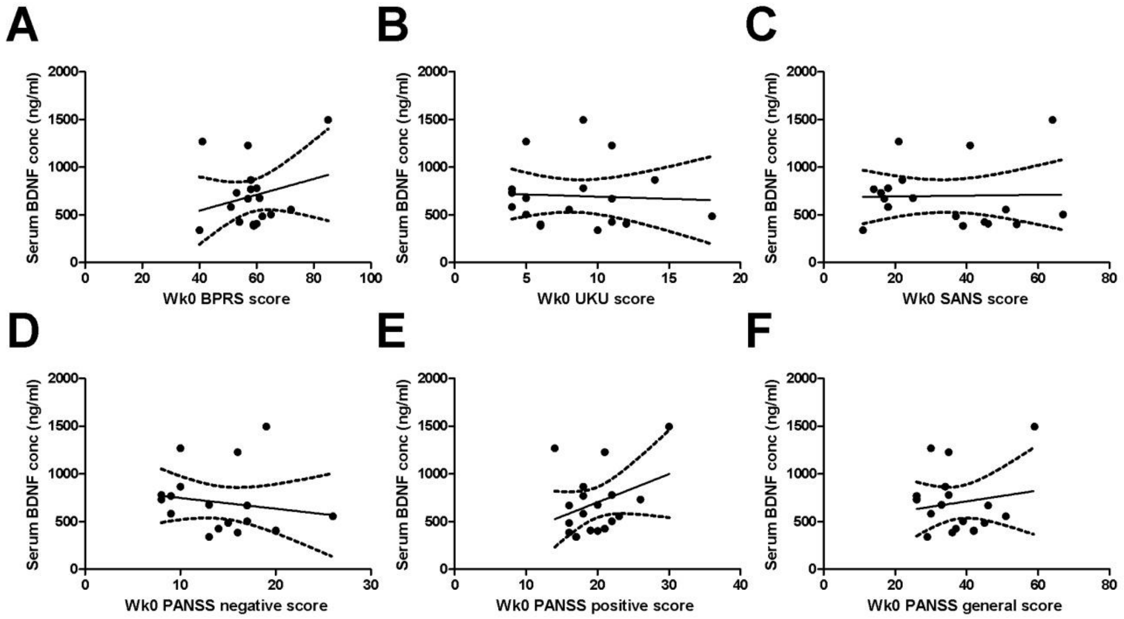

Supplementary Figure 1. Relationship of serum BDNF levels with outcome measures at week 0. Correlation analysis was performed for individual serum BDNF concentrations of FEP subjects with their corresponding scores for BPRS **(A)**, UKU **(B)**, SANS **(C)**, PANSS negative **(D)**, PANSS positive **(E)** and PANSS general **(F)**.

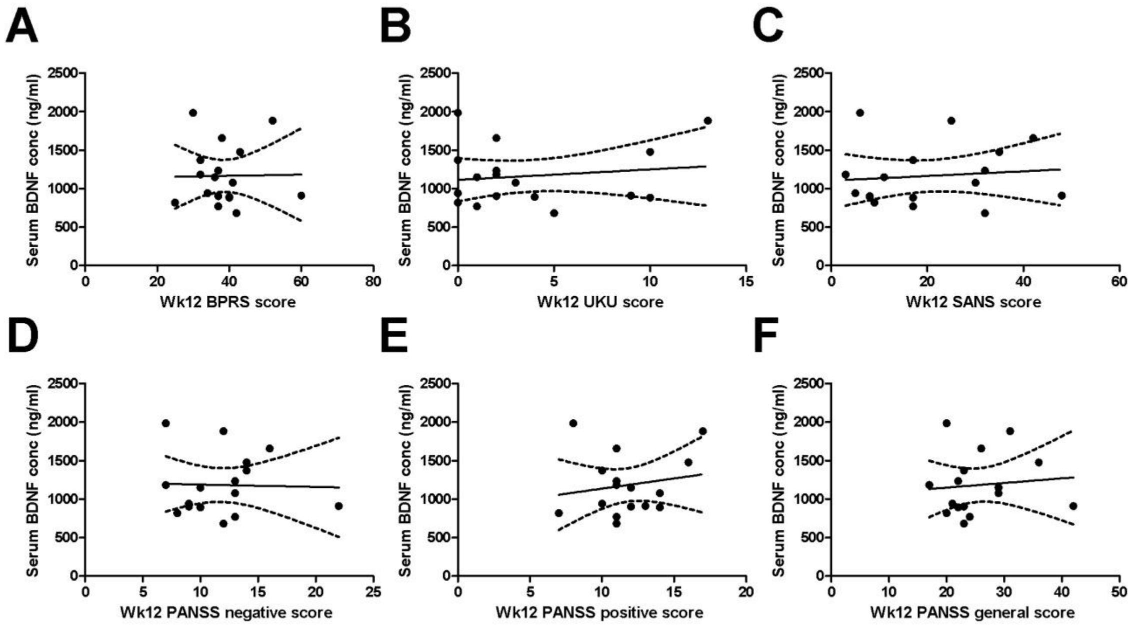

Supplementary Figure 2. Relationship of serum BDNF levels with outcome measures at week 12. Correlation analysis performed for individual serum BDNF concentrations of FEP subjects with their corresponding scores for BPRS **(A)**, UKU **(B)**, SANS **(C)**, PANSS negative **(D)**, PANSS positive **(E)** and PANSS general **(F)**.

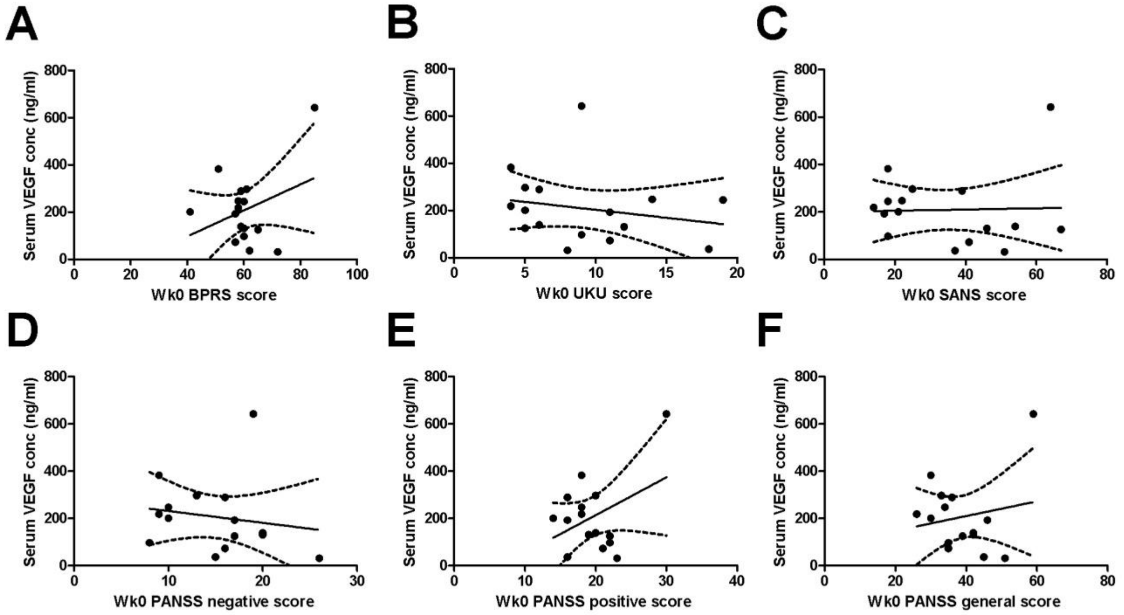

Supplementary Figure 3. Relationship of serum VEGF levels with outcome measures at week 0. Correlation analysis was performed for individual serum VEGF concentrations of FEP subjects with their corresponding scores for BPRS **(A)**, UKU **(B)**, SANS **(C)**, PANSS negative **(D)**, PANSS positive **(E)** and PANSS general **(F)**.

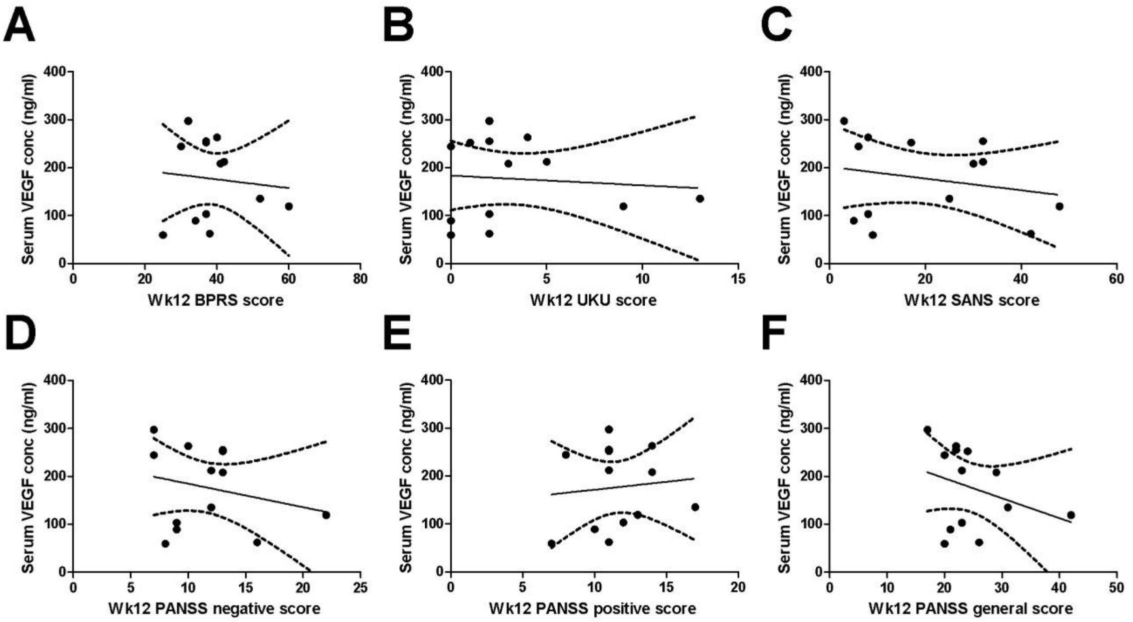

Supplementary Figure 4. Relationship of serum VEGF levels with outcome measures at week 12. Correlation analysis was performed for individual serum VEGF concentrations of FEP subjects with their corresponding scores for BPRS **(A)**, UKU **(B)**, SANS **(C)**, PANSS negative **(D)**, PANSS positive **(E)** and PANSS general **(F)**.
